# The Inferior Maxilla and Its Changes

**Published:** 1869-10

**Authors:** John R. Barr


### ARTICLE II.

### *The Inferior Maxilla and its Changes.*

By John R. Barr, D.D.S.

The physiological variations of this bone develop some of the most remarkable phenomena, that occur in the human organism during the formative process. No other bone, the superior maxilla excepted, undergoes so many changes.

Many of the mutations which the lower jaw is found actually to undergo, are the result of accident or disease, to both of which it is exposed from its prominent and isolated condition in the exercise of its functions. The loss of the teeth from decay is found to be the exciting cause of many of these effects. In a normal and unbroken condition of the economy, the teeth are retained in the jaw until old age, and we see no remarkable changes taking place except the slight approximation of the jaws, which results from the gradual wearing away of the crowns of the teeth by the mechanical act of mastication. Unfortunately, we rarely ever meet with such a condition, for where we find one jaw in old age still glistening with its pearly gems, we find fifty almost entirely devoid of them, or but with a few old blackened stumps—here and there—to remind us of its former usefulness. Disease being the general rule then, and health its exception, many of the variations of this bone are to be regarded as pathological effects.

We shall consider the teeth in their immediate relations to the lower maxilla, and the effects consequent upon their removal. During the first development and growth of the inferior maxillary bone, changes take place in its form and structure which have an important bearing upon the future existence of it, as a necessary and peculiar bone. When we see the first feint outlines of the jaw, these too are the first indications of dental development manifested, and these, upon the authority of Mr. Goodsir, can be seen as early as the fifth or sixth week of inter-uterine existence, when the human embryo is but an inch in length. Upon inspecting the cavity which we would scarcely at this early period call a mouth, we will see preparations for the dental organs in the primitive dental groove, which makes its appearance about this time, and from it the first stage of dental formation takes place. If, at the seventh week of inter-uterine existence, we again inspect the cavity, we will find on each side of the arch and at the bottom of the groove a single projection of mucous membrane; and this increases and after a short time becomes a papilla, which is the first and rudimentary form of the pulp of the tooth. In a few more weeks the papillæ of the temporary teeth are inclosed within the jaw, by the approach and closure of the lips of the primitive dental groove, in which we find them first formed. Coincident with the closing of the lips of the primitive dental groove, the provident maxilla opens another groove behind the first, in which are to be developed the permanent teeth. After the lapse of a few more weeks these in turn are shut in, and take their position behind the deciduous teeth. In this position their development is perfected, and and from this situation they grow and make their appearance through the gums, at a time when the child having grown and increased in vigor, stronger masticatory apparatus is required to triturate the more substantial food which the necessities of the system demand, as the child emerges from infancy to childhood, and from childhood to the full vigor of manhood. Thus we have the curious phenomena of one bone growing within another one, the jaw serving as a covering or matrix for the formation of another and lower order of being than itself. As soon as the teeth are fully formed, or nearly so, the bone makes its appearance loaded with those sparkling pearls of nature, which it has shielded from external injury, and at once begins the arduous duties which they are called upon to perform, in the active campaign of life. It is during this period, infancy, that the changes of this bone become most visible, and the mysterious result of the wonderful operations that nature has been carrying on in her unseen laboratory begin to show themselves. Just at the period when the growing wants of the child demand it, these gems of nature emerge from the bony parietes of the alveoli, and the soft coat of mucous membrane which covers them, their advance being heralded by pains which, like those of child-birth, announce the importance of those whom they precede. These organs have considerable to do just now with the configuration of the jaw, and this is shown by the premature extraction of a deciduous tooth, when the alveolar arch will contract, or it will remain stationary at that point, the base of the bone remaining in its original position and continuing to increase; this effect we readily conclude seems to be confined to the alveolar ridge. The jaw continues to increase in width and length during and even after the eruption of the deciduous teeth. This may be satisfactorily proven by the separation of the temporary incisors and canines, which phenomena is noticeable about the sixth or eighth year. And, somewhat later in life when the child emerges from childhood to the full bloom of manhood, a thickening and broadening of the anterior portion of it may be observed. We can easily show by the presence of the permanent molars behind the position occupied by the deciduous molars, which at the time of their eruption are found to be at the base of the coronoid processes, that there must have been an increase in the length of the lateral portions of the body of the jaw. The bone must have grown at this point between the coronoid processes and the temporary teeth. An increase of the body at the angles, as also an increase in the length of the rami, are likewise obvious and progressive. When the eruption of the permanent teeth has been completed, the rami are found to occupy nearly a vertical position with the plane of the bone, and were the teeth to remain in a normal condition, free from disease, and unaffected by the many accidents to which they are exposed, the jaw would be but little changed during adult life. The base of the jaw in the child is arched a little upwards, and backwards from the chin to the angle on either side, and this makes the bone at the base of the coronoid processes a little narrower than at any other point. In the adult this bone is of nearly the same breadth along its entire lateral length, as anatomists tell us, and in the aged person it becomes again arched, returning to almost its original direction after all the teeth are lost. Omitting those pathological changes of the bone in question, which are the result of irregularity of the teeth, we come to those changes which are brought about by the loss of the dental organs. The molar teeth from their large size and position in the arch, are the cause of many of the deformities of the jaw, they are generally the first to be attacked by disease, and, as a consequence, the first to fall a prey to the forceps. The bicuspids are the next upon which the leaden hand of decay is laid, and when we have the loss of both these classes of teeth, the change in the configuration of the jaw is almost always obvious.

The angle of the jaw then has no support against the powerful traction of the temporal and masseter muscles during the acts of mastication and deglutition. The bone, though strong and braced, is a living tissue and must yield; we now have the face of the young and perhaps once beautiful, expressing a premature old age.

The action of the muscles in producing this change may be seen when these conditions are examined. The under jaw when closed upon the upper, is in contact with it only at the anterior portion or chin, the condyle is articulated with the glenoid cavity, and between these extreme points there are two most powerful muscles, exerting great force upon an unsupported living tissue, and one endowed with but a small degree of elasticity. The effects of such action as this are frequently seen in persons of a lean, cadaverous look, having the plane of the lower maxilla at an enormous angle with the glenoid cavity, and with the front teeth in direct antagonism with those of the superior maxilla. The loss of the teeth commencing behind and progressing forward, the superior cuspidati and inferior incisors being the last to decay, this powerful traction of the temporal and masseter muscles continues through a long period, and when both jaws, having been denuded of teeth, come in contact with each other, the inferior maxilla will be found to have been elongated to such a degree, that the symphysis menti, will be thrown far beyond the anterior portion of the superior maxilla, and the face will present an unsightly appearance.

The absorption or gradual wasting away of the alveoli is another and the last of the eccentricities of this bone that I shall mention. So perfect is the change which it undergoes, that we would scarcely recognize in the smooth and rounded shape of it after absorption is completed, the bone of which we have been speaking. That both the outer and inner alveolar walls are absorbed upon the loss of the teeth, is a fact that I presume no dentist will deny, but in what manner this is effected, what peculiar agent is brought to bear, or how nature brings about such wonderful results, is at present uncertain. Many have been the theories advanced, and many have been the efforts made to account for this truly curious phenomena, but as yet none have been advanced that are altogether satisfactory. I may therefore without fear of being considered presumptious, theorize a little upon this point. Absorption we believe is effected by means of the blood-vessels, as is deposition; we may find proof that osseous substance is deposited by the blood-vessels, without going beyond the bone under consideration.

In the development of the teeth, that dense hard substance known as dentine, is deposited by a very vascular organ the pulp, at first the deposit is only a granular substance which afterwards becomes dentine. That absorption of bone is caused by an increased vascularity in the periosteum which surrounds it, we will endeavor to prove.

After the formation of the enamel is completed, the teeth commence to grow very rapidly, and they grow as we have been told in the lecture rooms, from their roots upwards, the crowns being first formed. Now as the crowns of these teeth are surrounded by very delicate membranes, it is fair to suppose that the pressure, which must be considerable, upon these delicate membranes as the teeth advance, produces a determination of blood to them. That the pressure is sufficient to produce such vascularity, may be shown by the phenomena so often witnessed in the eruption of the dentes sapientiæ, the gums are often inflamed and swollen, sometimes throbbing and giving great pain, these symptoms together with the extreme redness, show that there is an increased vascularity in the parts, caused by the pressure of the tooth beneath. We have then a vascular membrane, with its blood-vessels distended; and may not the action of these produce absorption of the alveolus at the point of pressure, and thus allow the tooth to issue from its bony prison, this is true in both first and second dentition; in the latter operation, the pulps of the deciduous teeth are affected, and under the same influence as the surrounding parts absorb the roots of their teeth. We believe that the periosteum is the agent through which the absorption of the alveolus is accomplished; it commences its work as soon as the teeth are removed. There is deposited in the sockets of the teeth, by the periosteum which lines them throughout their whole extent, an osseous substance This deposit commences at the bottom of the socket and continues until the whole is filled. We believe that superficial absorption, takes place by similar means under the same governing and directing power, until the alveoli are entirely obliterated. When the absorption of the process is completed, upon an examination we will find that the anterior plate has receded upon the inner one, giving a backward and upward slope to the external surface of the bone, and this, with the elevation resulting from the loss of the teeth and alveoli, gives a different position to the chin, and by this shortening of the face, and the approximation of the nose and chin, we recognize the characteristic expression of the aged person.

